# Study of a Long-Gauge FBG Strain Sensor with Enhanced Sensitivity and Its Application in Structural Monitoring

**DOI:** 10.3390/s21103492

**Published:** 2021-05-17

**Authors:** Jing Yang, Peng Hou, Caiqian Yang, Ning Yang

**Affiliations:** 1 Key Laboratory of Concrete and Prestressed Concrete Structures of the Ministry of Education, School of Civil Engineering, Southeast University, Nanjing 210096, China; yangjingseu@seu.edu.cn (J.Y.); houpeng@seu.edu.cn (P.H.); 2 College of Civil Engineering & Mechanics, Xiangtan University, Xiangtan 411105, China; 3 Shandong Institute of Space Electronics Technology, Yantai 264670, China; yn3714@163.com

**Keywords:** FBG strain sensor, long-gauge, enhanced sensitivity, sensitivity coefficient, structural monitoring

## Abstract

A long-gauge fiber Bragg grating (FBG) strain sensor with enhanced strain sensitivity is proposed, which is encapsulated with two T-shaped metal blocks. Its fabrication method is described briefly, and the strain sensitivity can be flexibly adjusted through changing its packaging method. A series of experiments are carried out to study the packaging and its sensing properties. The experimental results show that the strain and temperature sensitivity coefficient of the sensor are three times larger than the common FBG sensors. The linearity coefficients of the FBG sensor are larger than 0.999, and the relative error of the repeatability of all sensor samples is less than 1%. Through the stability test on the actual bridge, it is revealed that the long-term stability of the sensor is excellent, and the maximum error is less than 1.5%. In addition, the proposed FBG strain sensors are used to conduct a shear strengthening experiment on a reinforced concrete (RC) beam to verify its working performance. The experimental results show that the strain change and crack propagation of the RC beam are well monitored by the sensors during the loading process.

## 1. Introduction

The health monitoring technology of bridge structures has attracted widespread attention in the transport sector. The sensing technology is the hardware core of the health monitoring system and the technical basis for monitoring purposes. Therefore, the research and development of sensing technology is essential to achieve the consequent structural health monitoring (SHM).

At present, the sensors used for SHM in civil engineering mainly include vibrating wire sensors, resistance strain gauges and fiber Bragg grating (FBG) sensors [1,2,3,4,5,6]. The measurement accuracy of the vibrating wire sensor is relatively low, and it is easily affected by the magnetic field and surrounding environment. The resistance strain gauge is characterized by high measurement accuracy, a wide measuring range and a simple structure. However, it has a distinct non-linearity when faced with a large strain and weak signal. These shortcomings severely limit its application, especially in long-distance monitoring and harsh environments. FBG sensing technology has developed rapidly in recent years. It is characterized by high measurement precision, immunity to electromagnetic interference, stability of long-term sensing and so on. Therefore, the FBG sensing technology favors the long-term SHM [7,8,9].

However, the bare fiber grating is fragile and easily damaged when it is directly used as a sensor. Moreover, the bare fiber grating cannot be reused after installation on the bridge, which leads to a high cost. Therefore, in order to improve the durability and installation property of FBG, it is necessary to be encapsulated to reduce the erosion of the external environment of the fiber, such as ultraviolet and water vapor. The common encapsulation method of FBG sensors includes tube-encapsulated [10,11,12], fiber-reinforced polymer-encapsulated [13,14,15,16] and metal sheet-encapsulated methods [17,18].

At present, the “point” sensing method is commonly used in actual engineering structures. It is difficult to capture damage information for the large-scale civil engineering structure when using local detection with “point” sensors. In addition, it is difficult to detect minor damage by using “point”-type detection. Due to the various drawbacks of “point”-type detection, the concept of distributed long-gauge strain sensing was proposed and studied [5,15,19]. Generally, the strain of the bridge is relatively small, and it is difficult to achieve the purpose of accurate monitoring due to the smaller sensitivity coefficient of the ordinary FBG sensor. Therefore, it is necessary to develop an FBG strain sensor with high sensitivity. Some scholars have carried out research on the technology of distributed sensing and strain sensitization of FBG. Ren et al. [10] proposed an FBG strain sensor with enhanced strain sensitivity, which was encapsulated by two gripper tubes and was applied in the seismic tests of a small-scale dam model. Li et al. [15] developed an encapsulate method of FBG with basalt fiber-reinforced polymer, where the basic idea is to utilize two types of materials with different stiffness levels to package the in-tube optical fiber in order to impose the deformation within the gauge length largely on the short-gauge sensing part of FBG. Górriz et al. [20] presented a new long-gauge sensor for structural health monitoring based on the use of fiber Bragg gratings, whose principal novelty and advantage is that it does not require the prestressing of the optical fiber in order to function correctly. Yang et al. [21] performed a thorough analysis of the performance of different strain magnification structures with FBG. Through the conducted simulation and experiment, it was found that the proposed method could be used to enhance the sensitivity of the FBG sensor. Du et al. [22] proposed a method to enhance the strain sensitivity of an FBG sensor by degenerated four wave mixing for frequency chirp magnification, and the sensitivity of the sensor was 5.36 pm/με obtained by experiments. Guo et al. [23] described the structure design, parameter optimization and performance testing of an FBG strain sensor with features of surface-mounting and reusability, and the test results showed that the sensor provided a sensitivity of 3.357 pm/με with a fitting linear correlation coefficient of 0.9999.

In this paper, a long-gauge FBG strain sensor with enhanced strain sensitivity is proposed, which is encapsulated with two T-shaped metal blocks, and its fabrication method and sensitivity theory are studied as well. A series of calibration experiments are carried out to test its sensitivity coefficients of strain and temperature. In addition, the proposed FBG strain sensors are used for a shear strengthening experiment on a reinforced concrete (RC) beam to verify its performance.

## 2. Design of Sensitive FBG Strain Sensor

### 2.1. Design of the Sensor

**Figure 1:**
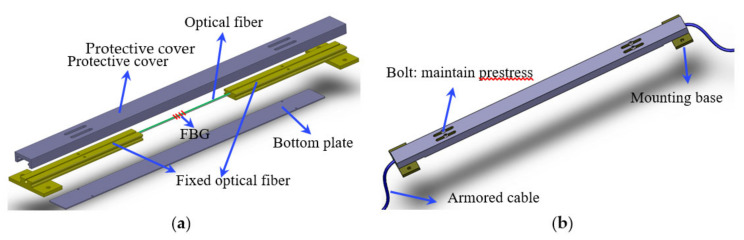
Structure of the sensor: (**a**) overall appearance of the sensor; (**b**) assembly drawing.

**Figure 2:**
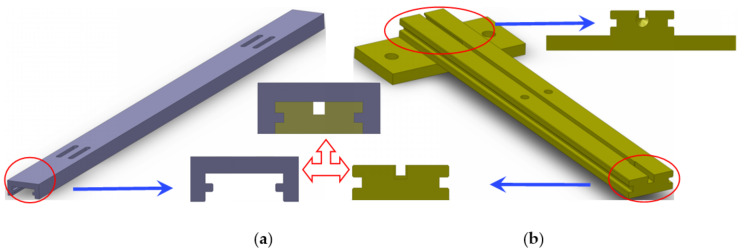
Structure diagram of the: (**a**) protective cover; (**b**) T-shaped metal block.

As shown in Figure 1, the sensor is composed of an FBG, two T-shaped metal blocks, an outer protective cover and a bottom plate, and it is also provided with an armored cable. The outer protective cover and bottom plate are used to protect the FBG, and armored flexible tubing protects the fiber pigtail. The optical fibers are fixed on T-shaped metal blocks, and they are also used as the mounting base in the installation process of the sensor. The outer protective cover and T-shaped metal blocks are assembled through the convex concave structure, and the metal block can slide along the inner side of the protective cover. The structure diagram is shown in Figure 2.

During the manufacture process of the sensor, the optical fiber needs to be pre-tensioned and fixed with glue in the groove of the metal blocks. The grating stays between two metal blocks. The applied prestress force to the optical fiber is maintained by bolts. This design feature greatly improves its measurement accuracy in the application process and makes it superior to other sensors. When the sensor is fixed on the structure by the mounting base, the bolts are loosened so that the metal block can slide freely; thus, the sensor can measure the strain data accurately. The measurement sensitivity of the sensor is much higher than that of the common FBG sensors. When the gauge length of the sensor is fixed, the strain sensitivity can be adjusted by changing the length of the T-shaped metal block or the anchoring length of the optical fiber (as better explained in Section 2.2). In addition, the metal package can provide efficient mechanical protection of the sensor, and it is convenient to install and disassemble. The gauge length of the sensor is determined by the distance between the mounting bases.

### 2.2. Theoretical Analysis of the Sensitization

**Figure 3:**
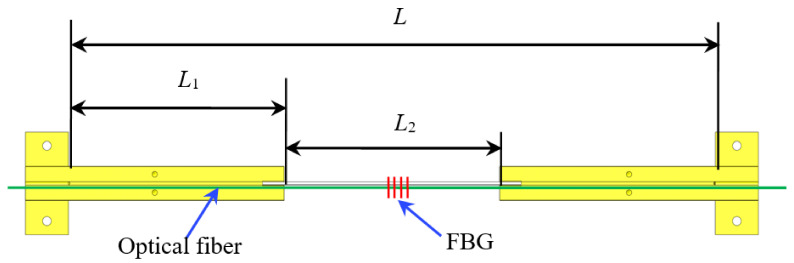
Schematic of sensor dimensions.

For the sensor, as shown in Figure 3, it is assumed that the gauge length of the sensor is *L*, the anchoring length of the optical fiber in the T-shaped metal block is *L*_1_, the distance between the two metal blocks is *L*_2_, and the elongation of the optical fiber under the tension *F* is Δ*L*. The strain *ε* can be expressed as below:

(1) $$\varepsilon =\frac{\Delta L}{L}$$

According to material mechanics:

(2) $$\Delta L=2\Delta L_{1}+\Delta L_{2}$$

in which Δ*L*_1_ is the elongation of the T-shaped metal block, and Δ*L*_2_ is the elongation of the optical fiber of *L*_2_. The strain *ε*_1_ of the metal block and the strain *ε*_2_ of the grating can be expressed as below:

(3) $$\varepsilon_{1}=\varepsilon_{2}=\frac{\Delta L_{1}}{L_{1}}=\frac{\Delta L_{2}}{L_{2}}$$

in which Δ*L*_1_ and Δ*L*_2_ can be expressed as:

(4) $$\left\{\begin{array}{c} \Delta L_{1}=\frac{FL_{1}}{E_{1}A_{1}}\\ \Delta L_{2}=\frac{FL_{2}}{E_{2}A_{2}} \end{array}\right.$$

in which *E*_1_ and *A*_1_ are the elastic modulus and sectional area of metal block; *E*_2_ and *A*_2_ are the elastic modulus and sectional area of optical fiber. Dividing Δ*L*_1_ by Δ*L*_2_, the following can be obtained:

(5) $$\frac{\Delta L_{1}}{\Delta L_{2}}=\frac{L_{1}E_{2}A_{2}}{L_{2}E_{1}A_{1}}$$

Taking the sensor with a gauge length of 30 cm as an example (*L* = 30 cm, *L*_1_ = *L*_2_ = 10 cm), and if the material of the metal is aviation aluminum alloy, the sectional area *A*_1_ of the metal block is 75 mm^2^, and the elastic modulus *E*_1_ is 70 GPa. The sectional area *A*_2_ and elastic modulus *E*_2_ of the optical fiber is 0.011 mm^2^ and 72 GPa, respectively. Submitting them into Equation (5), the following can be obtained:

(6) $$\Delta L_{1}\ll \Delta L_{2}$$

It can be seen from Equation (6) that the strain of the *L*_1_ section can be ignored compared with the strain of the *L*_2_ section. Therefore, it can be approximately considered that all the deformation of the sensor is concentrated in the *L*_2_ section of the optical fiber. Then, the strain *ε’* of the sensor can be approximately expressed as:

(7) $$\varepsilon^{\prime }\approx \frac{\Delta L}{L_{2}}$$

It is assumed that the strain enhancing coefficient of the sensor is *k*. When compared with the common FBG sensor, the coefficient *k* can be expressed as:

(8) $$k=\frac{\varepsilon^{\prime }}{\varepsilon }\approx \frac{\Delta L/L_{2}}{\Delta L/L}\approx \frac{L}{L_{2}}$$

Because *L* is greater than *L*_2_, the ratio is greater than 1. This is the basic principle of the sensitivity enhancement design. Therefore, the *k* is related to the ratio of the gauge length *L* of the sensor and the length of the *L*_2_ section. The strain sensitivity coefficient of the sensor can be adjusted by changing the anchoring length *L*_1_ of the optical fiber or the distance *L*_2_ between the two metal blocks.

## 3. Experimental Study on Sensing Performance

### 3.1. Strain Sensing Calibration

It is assumed that the strain enhancing coefficient of the sensor is *k*. Taking two kinds of commonly used sensors as an example, the gauge lengths of the sensors are *L* = 30 cm and *L* = 15 cm. The anchoring length of the optical fiber is *L*_1_, and the distance between the two metal blocks is *L*_2_. The strain sensitivity coefficient of a common FBG sensor or bare fiber is around 1.1 pm/με, and the maximum theoretical strain sensitivity coefficient of the sensor can be obtained according to Equation (8). The size specifications and strain sensitivity coefficients of the two kinds of sensors are shown in Table 1.

**Figure 4:**
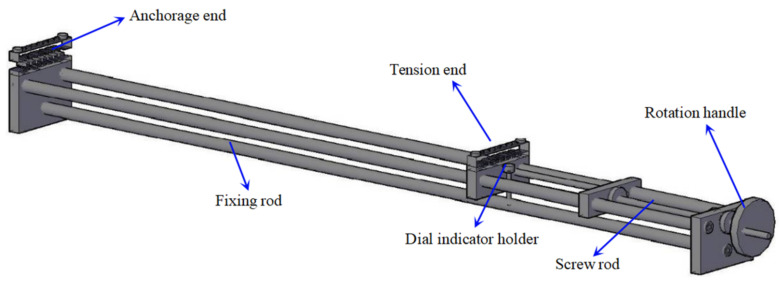
Structure of sensor calibration frame.

**Figure 5:**
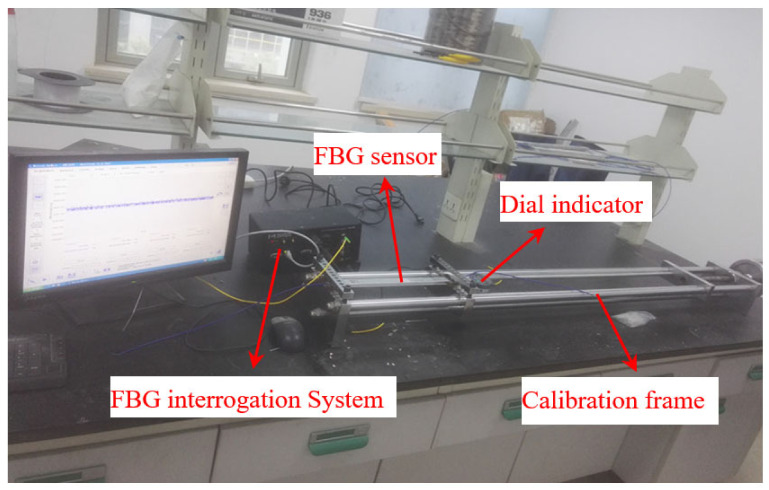
Process of calibration experiment.

**Figure 6:**
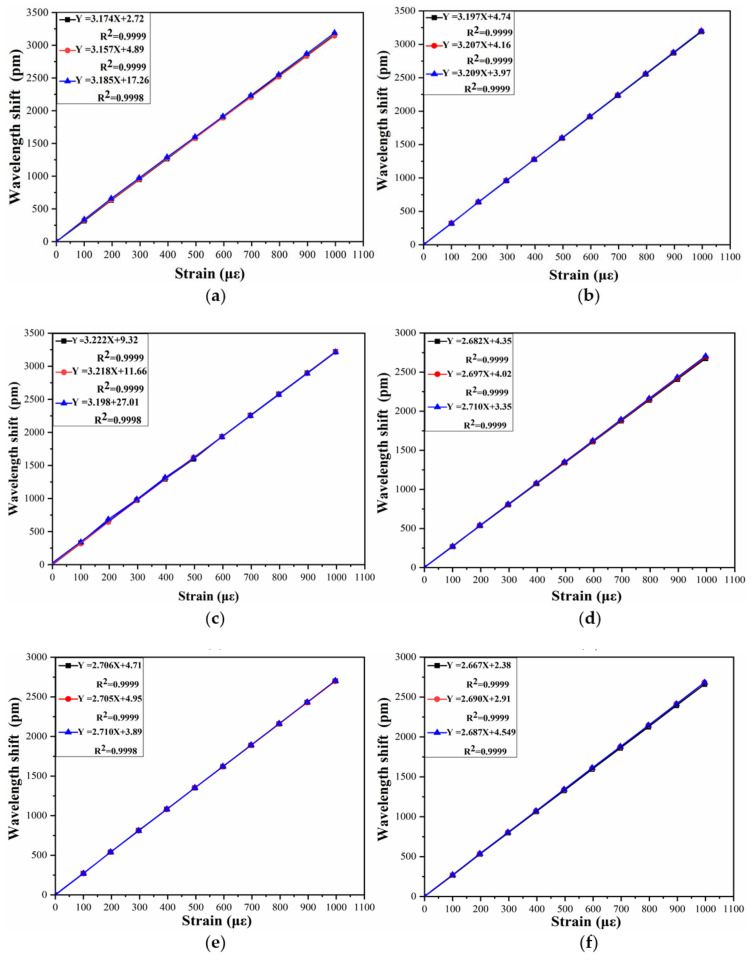
The calibration results of (a) FBG 1; (b) FBG 2;(**c**) FBG 3; (**d**) FBG 4; (**e**) FBG 5; (**f**) FBG 6.

**Figure 7:**
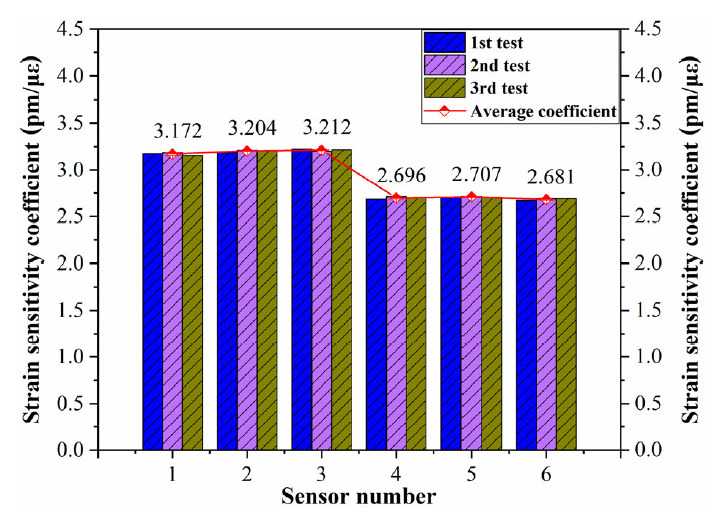
Repeatability results and test error of the FBG sensor.

Figure 4 shows the calibration frame for the calibration of long-gauge sensors. The calibration frame mainly solves the technical problems of batch calibration of long-gauge FBG sensors; it is characterized by high stiffness, good stability and excellent precision. The experimental set-up is shown in Figure 5. The wavelengths of the FBG sensors are acquired with MOI’s S130 model acquisition instrument, and the sampling rate is 500 Hz. Six sensors are selected for the calibration test, with two different gauge lengths, as shown in Table 2. Each sensor is pre-tensioned with different levels before the experiment and tested three times. The calibration results are shown in Figure 6. It can be seen that the average strain sensitivity coefficients of sensors FBG1-FBG3 are 3.172 pm/με, 3.204 pm/με and 3.212 pm/με, respectively. The strain sensitivity coefficients of sensors FBG4–FBG6 are 2.696 pm/με, 2.707 pm/με and 2.681 pm/με, respectively. The linearity coefficients of the FBG sensors are greater than 0.999. Figure 7 shows the repeatability results and test error of the FBG sensor. It can be seen that the relative error of the repeatability for all tested sensors is less than 1%. Moreover, the testing error of the same specification sensor is less than 1.5%, which is within an acceptable range. Therefore, the measurement accuracy of the proposed FBG sensor is less than 0.5με as the resolution of the data acquisition instrument is 1 pm.

The error sources should be analyzed in the following aspects: (1) the error of anchorage length during the manufacture process of the sensor; (2) a possible small bond slip between the optical fiber and the metal anchor block; (3) the ambient noise and temperature affect the test result to a certain extent; (4) the reading error of acquisition instrument and dial indicator.

### 3.2. Long-Term Sensing Stability

**Figure 8:**
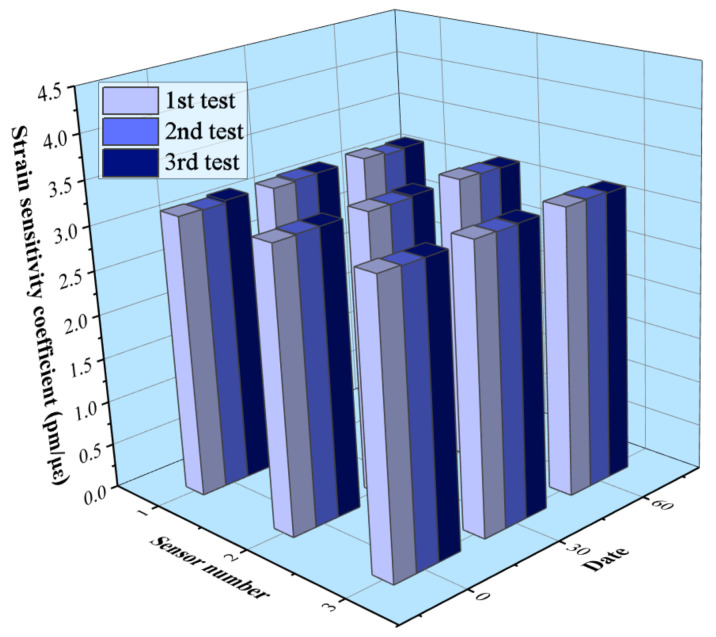
Long-term stability of the FBG sensor.

In order to study the long-term stability performance of the proposed FBG sensor, three sensors with a gauge length of 30 cm are installed on a small bridge. The strain sensitivity coefficient is tested for the first month and the third month, and the results are shown in Figure 8. It can be seen that the strain sensitivity coefficients of the samples are between 3.15 pm/με and 3.2 pm/με, and the maximum test error is less than 1.5%. Moreover, the strain coefficient error of a single sensor is less than 1% in the long-term test. The results indicate that the proposed FBG sensor has good long-term measurement stability.

### 3.3. Temperature Sensing Calibration

**Figure 9:**
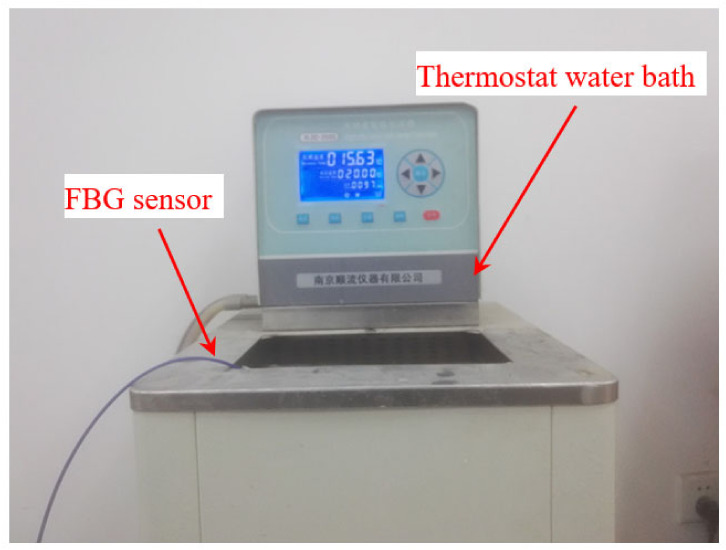
Temperature coefficient test of the FBG sensor.

**Figure 10:**
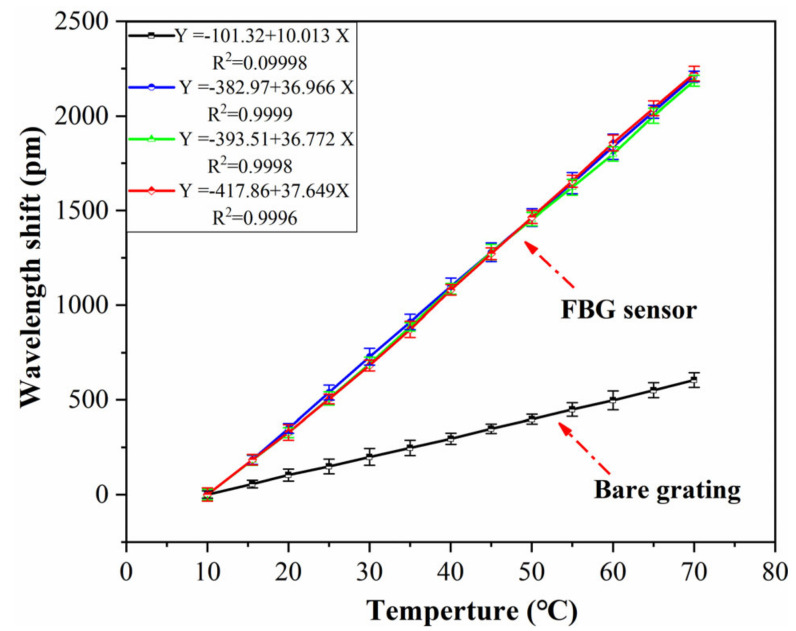
Test results of temperature coefficient.

The temperature is an important factor affecting the sensing performance of an FBG sensor because the FBG is sensitive to changes in both strain and temperature. Therefore, it is necessary to consider the influence of temperature in practical engineering, and the temperature sensitivity coefficient should be determined. The strain sensor in this paper is encapsulated by a metal block. The thermal expansion coefficient of the metal is larger than that of the optical fiber, so the FBG sensor also has the effect of temperature sensitization. The temperature sensitivity coefficient of the sensor is calibrated by the method of water bath heating (as shown in Figure 9). The temperature range is set to 10–70 °C, and the data are recorded every 5 °C. Three FBG sensors are selected to be calibrated in the experiment, and the test results with the error bar are shown in Figure 10.

It can be seen that the temperature sensitivity coefficient of the bare grating is 10.01 pm/°C, and the correlation linearity is 0.9998. The temperature sensitivity coefficients of the selected sensors are 36.96 pm/°C, 36.77 pm/°C and 37.64 pm/°C, around three times that of the bare grating. The correlation linearity of the FBG sensor is higher than 0.999, and the relative error of different samples is less than 3%, which indicates good accuracy and stability.

### 3.4. Repeatable Performance Test

**Figure 11:**
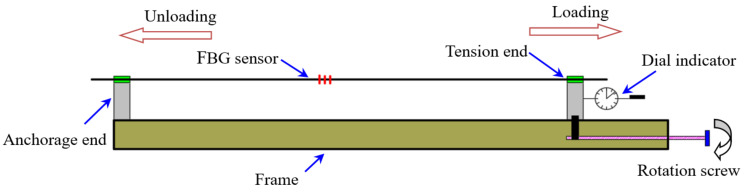
Repeatability test of the sensor.

The load of the actual engineering structures has many uncertainties, and the monitored parts may be affected by the tension and compression changes, or by the alternating tension and compression changes. The hysteresis of the sensor is an important parameter. Therefore, it is necessary to verify the repeatability of the sensor under a repeated load. The repeatability test of the sensor is carried out on the calibration frame, as shown in Figure 11. The sensor is loaded and unloaded repeatedly to test its wavelength variation.

**Figure 12:**
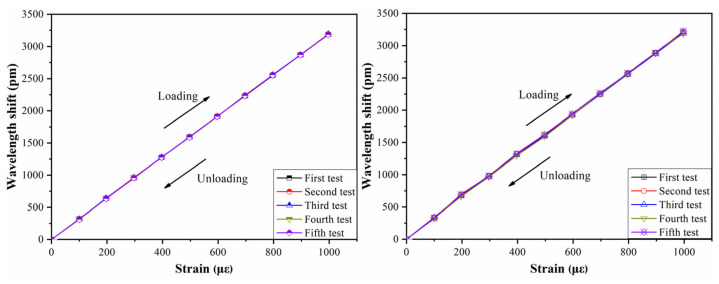
The results of repeatability test.

Two sensors are selected to be tested, and each sensor is tested 5 times. The results are shown in Figure 12. It can be seen that the strain wavelength variation curves of each sensor basically coincide with the same slope after 5 cycles of the loading and unloading process. The wavelength variation of the sensor returns to zero again after each unloading, thus showing that the FBG sensor has stable performance.

### 3.5. Study on Temperature Self-Compensation of Strain Sensor

Because the FBG is sensitive to changes in both strain and temperature, it brings the cross-sensitivity of temperature and strain to the actual measurement. Therefore, eliminating the influence of temperature is the basis of accurate measurement of strain. At present, most of the temperature compensation methods use an additional temperature sensor on the structure as a reference. However, the bridge structure is usually large in scale, and there are temperature gradients. Therefore, the temperature compensation accuracy is low and the error is large, which is not conducive to popularization and application.

**Figure 13:**
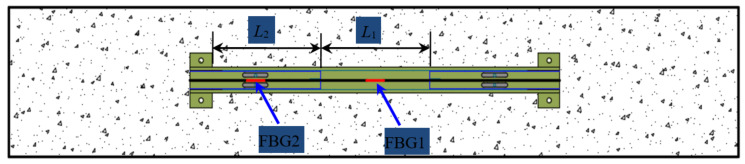
Schematic diagram of temperature compensation.

On the basis of the long-gauge strain sensor introduced above, the cross-sensitivity decoupling method of strain and temperature is studied for temperature compensation. As shown in Figure 13, two Bragg gratings (FBG1 and FBG2) are engraved on the fiber. Here, FBG2 is encapsulated in the groove of the T-shaped metal block (*L*_2_ length), and the stiffness of *L*_2_ is much greater than that of *L*_1_, so the wavelength change in FBG2 is basically caused by the temperature change. Therefore, the cross-decoupling method of strain and temperature can be established by Equation (9).

(9) $$\varepsilon =\left[\left(\lambda_{S}-\lambda_{1}\right)-\left(\lambda_{T}-\lambda_{2}\right)\cdot \frac{K_{S-T}}{K_{T}}\right]\cdot K_{S}$$

in which *ε* is the strain of the measuring point, *λ_S_* is the wavelength measured by FBG1, *λ*_1_ is the wavelength of FBG1 at T_0_, *λ_T_* is the wavelength measured by FBG2, *λ*_2_ is the wavelength of FBG2 at T_0_, *K_S-T_* is the temperature coefficient of FBG1, *K_T_* is the temperature coefficient of FBG2, and *K_S_* is the strain coefficient of FBG1.

**Figure 14:**
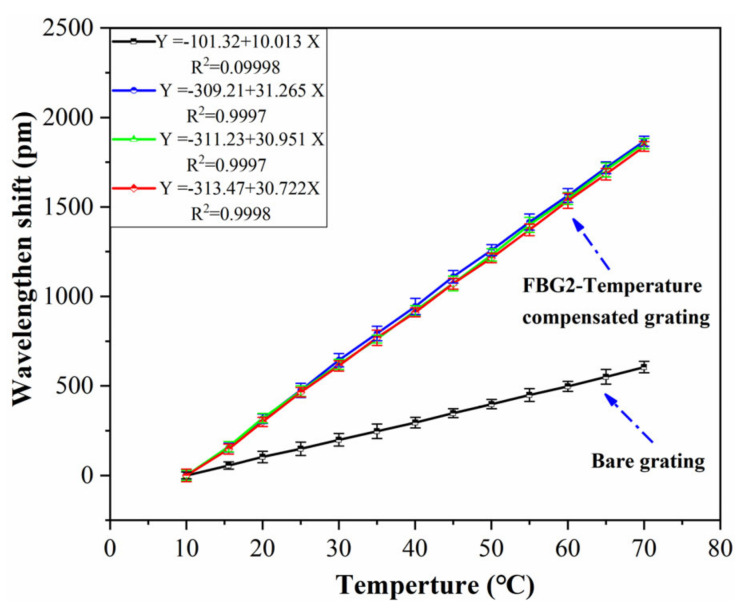
Test results of temperature self-compensation method.

In order to verify the temperature-compensation method, three sensors are selected for the calibration experiment. The water bath heating method is also used in the experiment. The temperature range is set to 10–70 °C, and the data are recorded every 5 °C. The results are shown in Figure 14. The results showed that the temperature sensitivity coefficients of the temperature-compensated grating of the selected sensor sample (FBG2) are 31.26 pm/°C, 30.95 pm/°C and 30.72 pm/°C, respectively, which is three times that of the bare grating. The relative linearity is greater than 0.999, and the relative error of the sensor samples is less than 2%.

## 4. Structural Monitoring of Strengthened RC Beams

### 4.1. Experimental Setup

**Figure 15:**
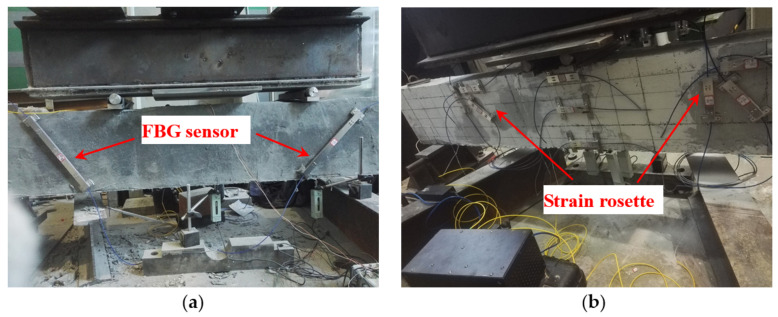
Layout of the sensors: (**a**) oblique sensors; (**b**) strain rosettes.

In order to research the effect of different reinforcement thicknesses of engineered cementitious composite (ECC) on the shear behavior of RC beams, three beams are manufactured in the experiment. The beam is 2100 mm in length, and the section size is 150 mm × 300 mm. One of the beams is the contrast beam, and the experimental beams are thickened with ECC, and the thickness is 2 cm and 4 cm, respectively. The experiment used the four-point loading method, and the loading device is a 100-ton hydraulic servo testing machine. The loading rate of each stage is set at 10 kN, and the load is kept for around 5 min. The development of cracks is observed after each loading condition. The proposed FBG strain sensors presented in this paper are used to monitor the strain change and crack development of the beam in the loading process. Two long-gauge sensors of 30 cm are installed on one side of the beam to monitor the crack, and the direction of the sensors is inclined by 45° near the loading point, as shown in Figure 15a. In addition, 6 FBG sensors with a gauge length of 15 cm are installed as an oblique 45° three-direction strain rosette, as shown in Figure 15b.

### 4.2. Experimental Results

#### 4.2.1. Comparison of Middle-Span Strain

**Figure 16:**
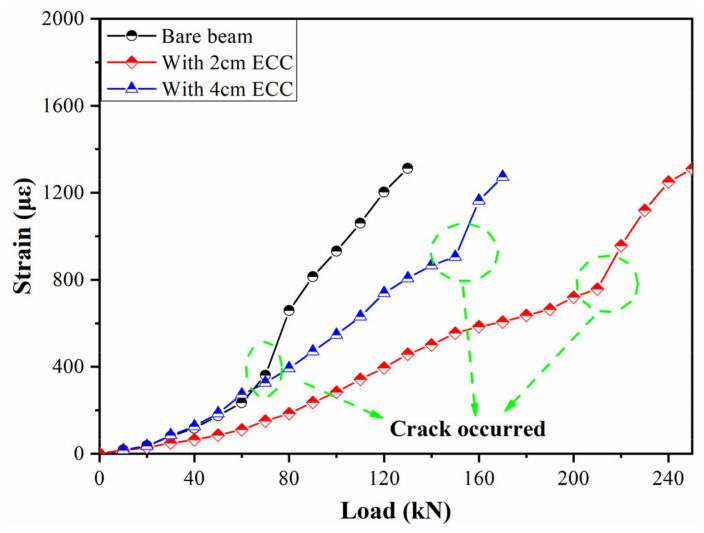
Load–strain curve.

The bearing capacity of the beam can be evaluated by comparing the middle-span strain of the beam bottom under loading test. The strain change is calculated by the wavelength and strain sensitivity coefficient of the FBG sensor. The middle-span load–strain curve of the three beams is shown in Figure 16. It can be seen that the strains of the three beams are almost the same before the load of 40 kN. However, the bottom strain of the contrast beam increases when the load is greater than 40 kN. The strain increases sharply when the load reaches 60–80 kN. It is speculated that there are cracks in the range of the sensor monitoring, and this was confirmed by field inspection. In addition, the strain change is more stable for the beams reinforced by ECC. The strengthening effect is more obvious, especially in the case where the ECC is 2 cm. The cracks occurred when the force reached up to 150 kN for the beam with 4 cm ECC.

#### 4.2.2. Monitoring of the Development of Oblique Cracks

**Figure 17:**
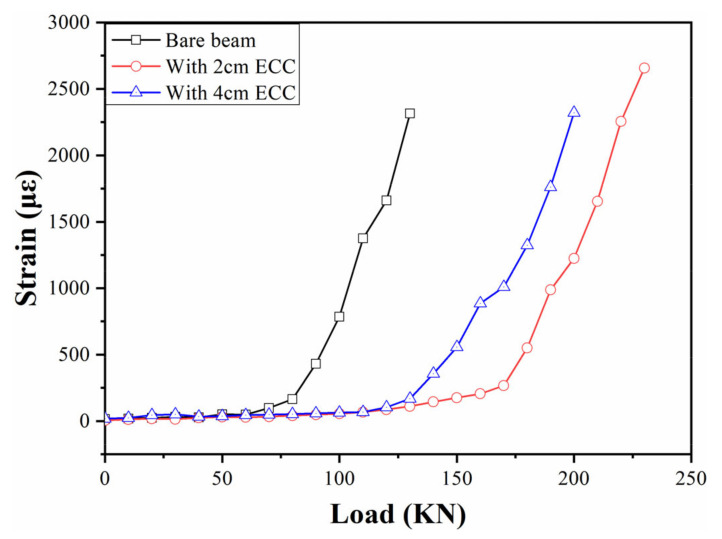
Oblique crack monitoring.

**Figure 18:**
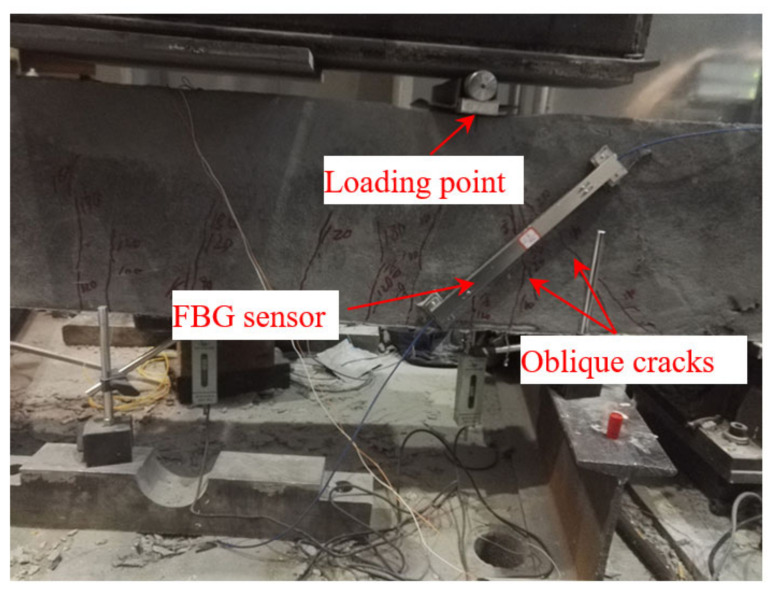
Monitoring of oblique cracks.

Oblique cracks appear near the loading point in the process of loading, which is the main failure mode of concrete beams under bending moment, and the cracks increase with the increase in load. Therefore, the formation and development of oblique cracks can be used to judge the working performance of the concrete beam. In the experiment, the oblique cracks are monitored by the FBG strain sensors with a gauge length of 30 cm, and the laying position is perpendicular to the direction of the development of the oblique cracks. The results are shown in Figure 17. For the bare beam, it can be seen that the strain increases slowly with the loading process. However, the strain increases sharply when the load reaches 80 kN, which is the manifestation of the rapid development of cracks in the sensor monitoring area (as shown in Figure 18). However, the cracking loads are 140 kN and 180 kN for the beams strengthened with 2 cm and 4 cm ECC, respectively. Therefore, the cracking load of the beam strengthened with ECC is greater than the bare beam, and the overall change trend is more stable.

#### 4.2.3. Monitoring of Shear Strain

**Figure 19:**
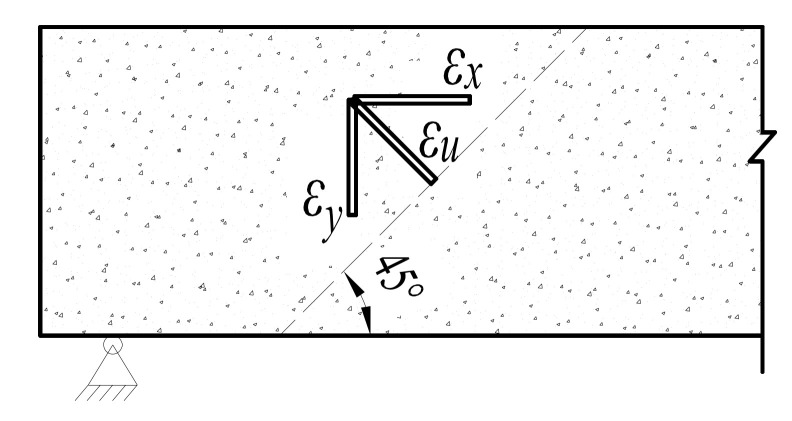
Schematic diagram of three-direction strain rosette.

**Figure 20:**
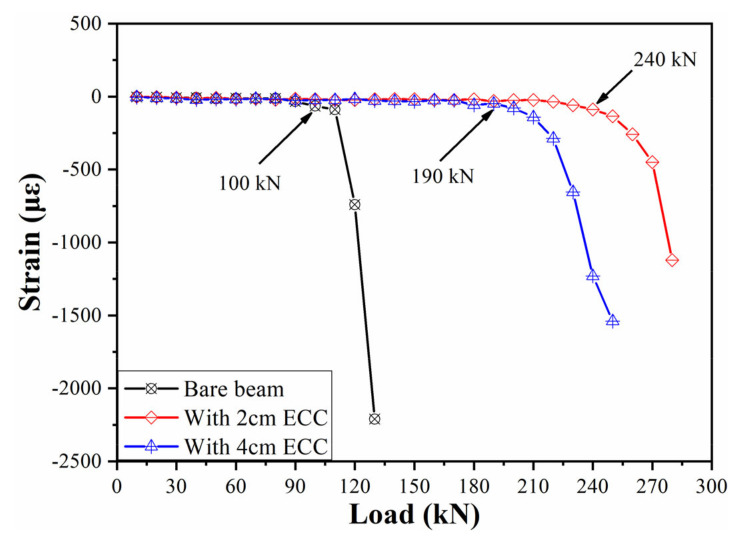
Monitoring of the shear strain.

The generation and development of oblique cracks are the main signs of shear failure of the beam. The oblique 45° three-direction strain rosettes composed of FBG strain sensors are arranged at the expected position of the inclined crack, which is the connecting line between the loading point and support. The development of an oblique crack and the change in beam structure are analyzed by monitoring the shear strain. The position of the strain rosettes is shown in Figure 15b, and the schematic diagram of strain rosettes is shown in the Figure 19. Assuming that the strains monitored by each sensor are *ε_u_*, *ε_x_*, *ε_y_*, respectively, the shear strain of the monitoring area could be calculated by the Equation (9). The results of shear strain are shown in Figure 20. It can be seen that the corresponding loads of the three beams are 100 kN, 240 kN, 180 kN, respectively, when the shear failure occurs. Therefore, the process of shear failure is more stable for the beam strengthened with ECC.

(10) $$r=\varepsilon_{x}+\varepsilon_{y}-2\varepsilon_{u}$$

## 5. Conclusions

A long-gauge FBG strain sensor with enhanced strain sensitivity is proposed. A series of experiments are carried out to study the sensing properties and performance. The main conclusions are as follows:

1. Compared with common commercial FBG sensors, the strain sensitivity coefficient of the proposed FBG sensor is greatly enhanced. Its strain sensitivity coefficient is 3.2 pm/με for the sensors with a gauge length of 30 cm, and the correlation linearity is greater than 0.999. Moreover, the design theory indicates that its strain sensitivity coefficient can be adjusted flexibly by changing its packaging method.
2. The temperature test showed that temperature sensitivity coefficient of the FBG sensor is around three times that of the bare grating, and the relative error of different samples is less than 3%. The hysteresis experiment shows that the proposed sensor has stable performance.
3. The proposed FBG strain sensors are used for a shear strengthening experiment on an RC beam to verify its working performance. The experimental results show that the strain change and crack development are well monitored by the FBG sensors during the loading process, and the beam strengthened by 2 cm ECC has the better effect.

## Figures and Tables

**Table 1:** The size specifications and strain sensitivity coefficients.

Gauge Length *L* (cm)	*L*_1_ (cm)	*L*_2_ (cm)	Strain Enhancing Coefficient *k*	Theoretical Strain Coefficient *K* (pm/με)
30	10	10	3	3.30
10	3	4	2.5	2.75

**Table 2:** Information of the test sensor.

Gauge Length *L*	30 cm			15 cm		
Sensor number	1	2	3	4	5	6
Wavelength (pm)	1542	1545	1556	1530	1549	1565

## Data Availability

Not applicable.
